# Hybrid-type and two-tetrad antiparallel telomere DNA G-quadruplex structures in living human cells

**DOI:** 10.1093/nar/gkz276

**Published:** 2019-04-12

**Authors:** Hong-Liang Bao, Hong-shan Liu, Yan Xu

**Affiliations:** Division of Chemistry, Department of Medical Sciences, Faculty of Medicine, University of Miyazaki, 5200 Kihara, Kiyotake, Miyazaki 889-1692, Japan

## Abstract

Although the telomeric sequence has been reported to form various G-quadruplex topologies *in vitro* and in *Xenopus laevis* oocytes, in living human cells, the topology of telomeric DNA G-quadruplex remains a challenge. To investigate the human telomeric DNA G-quadruplex in a more realistic human cell environment, in the present study, we demonstrated that the telomeric DNA sequence can form two hybrid-type and two-tetrad antiparallel G-quadruplex structures by in-cell ^19^F NMR in living human cells (HELA CELLS). This result provides valuable information for understanding the structures of human telomeric DNA in living human cells and for the design of new drugs that target telomeric DNA.

## INTRODUCTION

Human telomeric DNA exists at the end of chromosome and has important functions in cancer and aging (1–3). Telomere DNA consists of a duplex region with d(GGGTTA) repeats and a single strand 3′-overhang of 100–200 bases. Four repeats of the TTAGGG sequence have been reported to fold into intramolecular G-quadruplex structure (4,5). The G-quadruplex formation by telomeric DNA was demonstrated to inhibit the activity of telomerase. The enzyme telomerase is activated in 80% of human cancer cells (6,7). This makes telomerase and telomere DNA present a target with good selectivity for tumors. Thus, the biological importance of telomeric DNA G-quadruplex lets it become attractive structures for anticancer drug design (8–10). Numerous approaches have been developed to targeting telomeric DNA G-quadruplex structure (11).

Structural investigations have shown that telomere sequences form G-quadruplexes with various folding topologies (1). For example, a 22-mer human telomeric sequence d[AGGG(TTAGGG)_3_] was reported to form a basket-type antiparallel G-quadruplex structure in Na^+^ solution (12), while it primarily forms the hybrid-1 and hybrid-2 G-quadruplex structures in K^+^ solution (13–16). The same 22-mer sequence was also indicated to adopt a completely different parallel G-quadruplex structure in a crystal grown in the presence of K^+^ ions, as well as molecular crowding mimics (17,18). Furthermore, another telomeric sequence d[GGG(TTAGGG)_3_T] was reported to adopt an antiparallel basket type G-quadruplex with only two G-tetrad layers in K^+^ solution (19–21). Recently, some small molecules and antibodies have been used to investigate DNA G-quadruplex structures in living cells (22,23). However, these methods cannot distinguish the different topologies of G-quadruplexes. Very recently, as we prepared this manuscript, a paper by Srivatsan *et al.* appeared online and reported that telomeric DNA sequences can form a hybrid-2 G-quadruplex structure in *Xenopus laevis* oocytes (24) as an animal cell model. Recently, we employed ^19^F NMR spectroscopy to investigate human telomere RNA structures *in vitro* and in *Xenopus laevis* oocytes (25,26). It was demonstrated that ^19^F NMR spectroscopy can distinguish different nucleic acid structures by the corresponding ^19^F signal (27–31).

Although these approaches gave some structural information, the topology of telomeric DNA G-quadruplex present in living human cells has not yet been obtained. To investigate the human telomeric DNA G-quadruplex in a more realistic human cell environment, we performed the in-cell ^19^F NMR experiment in HELA CELLS, which is a big step forward to assess the structure of human telomeric DNA in living human cells. We demonstrated for the first time that the telomeric DNA sequence forms the hybrid-1, hybrid-2 and two-tetrad antiparallel G-quadruplex structures in living human cells.

## MATERIALS AND METHODS

### Sample preparation

3,5-bis(trifluoromethyl)phenyl moiety was incorporated into the 5′ terminal of DNA on a 1.0 μmol scale using an automatic DNA synthesizer and solid-phase phosphoramidite chemistry. After DNA synthesis, the oligomers were detached from the support, deprotected and purified by HPLC with an appropriate linear gradient of 50 mM ammonium formate in H_2_O and 50 mM ammonium formate in 1:1 acetonitrile/H_2_O. The oligomers were desalted by NAP 10 column (GE Healthcare) and identified by MALDI-TOF-MS on an autoflex III smart beam mass spectrometer (negative mode).

### Introduction of ^19^F-labeled DNA into HeLa cells by streptolysin O (SLO) treatment

HeLa cells grown in Dulbecco’s modified Eagle’s medium (DMEM) medium containing 10% FBS under a 5% CO_2_ atmosphere were harvested (2 × 10^7^) and washed twice with Hanks' Balanced Salt Solution (HBSS) buffer. Streptolysin O (Bioacademia) was activity with 10 mM DTT and 0.05% bovine serum albumin at 37°C for 2 h. To form pores on plasma membranes of HeLa cells, the activity SLO was added into HeLa cells with a final concentration of 0.1 μg/ml. Then, the HeLa cells were incubated at 4°C for 15 min with gentle rotation. The cells were washed three times with ice-cold HBSS buffer, followed by incubation with 3 mM ^19^F-labeled DNA in 500 μl HBSS buffer at 37°C for 30 min. For resealing of the HeLa cells membranes, CaCl_2_ was added to a final concentration of 1 mM and the HeLa cells were incubated at 37°C for 30 min. The HeLa cells were washed three times by HBSS buffer containing 1 mM CaCl_2_. The resealed HeLa cells were layered onto HBSS buffer containing 14% percoll (precentrifuged at 2000 × *g* for ∼60 min), and then centrifuged at 400 × *g* for 3 min. The cell pellet (living cells) was washed three times with HBSS buffer after the centrifugation.

### CD measurement

CD spectra were recorded using a Jasco model J-820 CD spectrophotometer and a 1 cm path length cell. About 0.3 ml samples at a 5 μM strand concentration in the presence of 100 mM KCl, 20 mM K-PO_4_ buffer (pH 7.0) or 300 mM NaCl, 20 mM Na-PO_4_ buffer (pH 7.0) were prepared for CD measurement. The natural and ^19^F-labeled oligonucleotides samples were heated at 95°C for 5 min and gradually cooling to 20°C.

### ^19^F NMR experiments

For *in vitro*^19^F NMR measurement, DNA samples of 100 μM concentration were dissolved in 150 μl of designed solvent containing 10% D_2_O, 300 mM NaCl and 20 mM Na-PO_4_ buffer (pH 7.0), or 100 mM KCl and 20 mM K-PO_4_ buffer (pH 7.0). Samples were prepared by heating the ^19^F-labeled oligonucleotides at 90°C for 3 min and gradually cooling to room temperature. ^19^F NMR spectra were recorded at a frequency of 376.05 MHz on a Bruker AVANCE 400 MHz spectrometer. CF_3_COOH was referenced as an internal standard (−75.66 ppm). Experimental parameters were as follows: spectral width 89.3 kHz, ^19^F excitation pulse 15.0 μs, relaxation delay 1.5 s, acquisition time 0.73 s, number of scans 256, line broad 3. Biomolecules concentration in living cells that introduce by SLO method is detected by comparing the fluorescence intensity of in-cells and *in vitro* samples according to previous study (32). Using 1 mM fluorescent DNA could result in a 50 μM final concentration in living cells. For in-cell ^19^F NMR, 3 mM DNA sample was incubated with the HeLa cells. Thus, the intracellular concentration of the ^19^F-labeled DNA should be around 150 μM according to previous study.

For in-cell ^19^F NMR measurement, SLO-treated HeLa cells were suspended in 200 μl of DMEM with 15% D_2_O. After the in-cell measurement, the cell suspension was supplemented with 100 μl of DMEM, and the supernatant was collected by centrifugation at 400 *g* for 3 min. The NMR spectrum of the supernatant was measured with the same number of scans 2048 as the in-cell NMR measurement.

### ^1^H NMR experiments

For 1D ^1^H NMR measurement, DNA samples of 0.5 mM concentration were dissolved in 150 μl of designed solvent containing 10% D_2_O, 300 mM NaCl and 20 mM Na-PO_4_ buffer (pH 7.0), or 100 mM KCl and 20 mM K-PO_4_ buffer (pH 7.0). Samples were prepared by heating the oligonucleotides at 90°C for 3 min and gradually cooling to room temperature. For 2D NOESY NMR measurement, DNA samples of 5.0 mM concentration were dissolved in 150 μl of designed solvent containing 10% D_2_O, 300 mM NaCl and 20 mM Na-PO_4_ buffer (pH 7.0). The mixing time was 200 ms.

### Fluorescence microscopy

The resealed cells were visualized with a TCS SP8 confocal microscopy (Leicamicro systems). The data were recorded using Leica software.

### Flow cytometry analysis

The resealed cells were resuspended in HBSS buffer containing 5 μg/ml propidium iodide (PI). The prepared cells were subjected to flow cytometry using an BD FACSC Calibur Flow Cytometer (BD Biosciences).

## RESULTS AND DISCUSSION

### *In vitro* ^19^F NMR analysis of ^19^F-labeled telomeric DNA G-quadruplexes

Telomeric DNA sequences were reported to form various G-quadruplex in different conditions (12–21). To study the telomeric DNA structure in a more native-like molecular crowding condition, Hansel *et al.* employed 1D ^1^H NMR and 2D ^1^H−^15^N sfHMQC NMR to investigate telomeric conformation in *Xenopus laevis* oocytes (21). Recently, 1D ^19^F NMR spectroscopy was used as a powerful tool to study telomeric DNA and RNA structures *in vitro* and in *Xenopus laevis* oocytes (24–26). However, the structure of telomeric DNA in human cells remains a challenge. Here, in order to directly identify the topology of telomeric DNA G-quadruplex in human cells, we characterized the conformation of several telomeric sequences using ^19^F NMR spectroscopy in HeLa cells.

**Figure 1.**
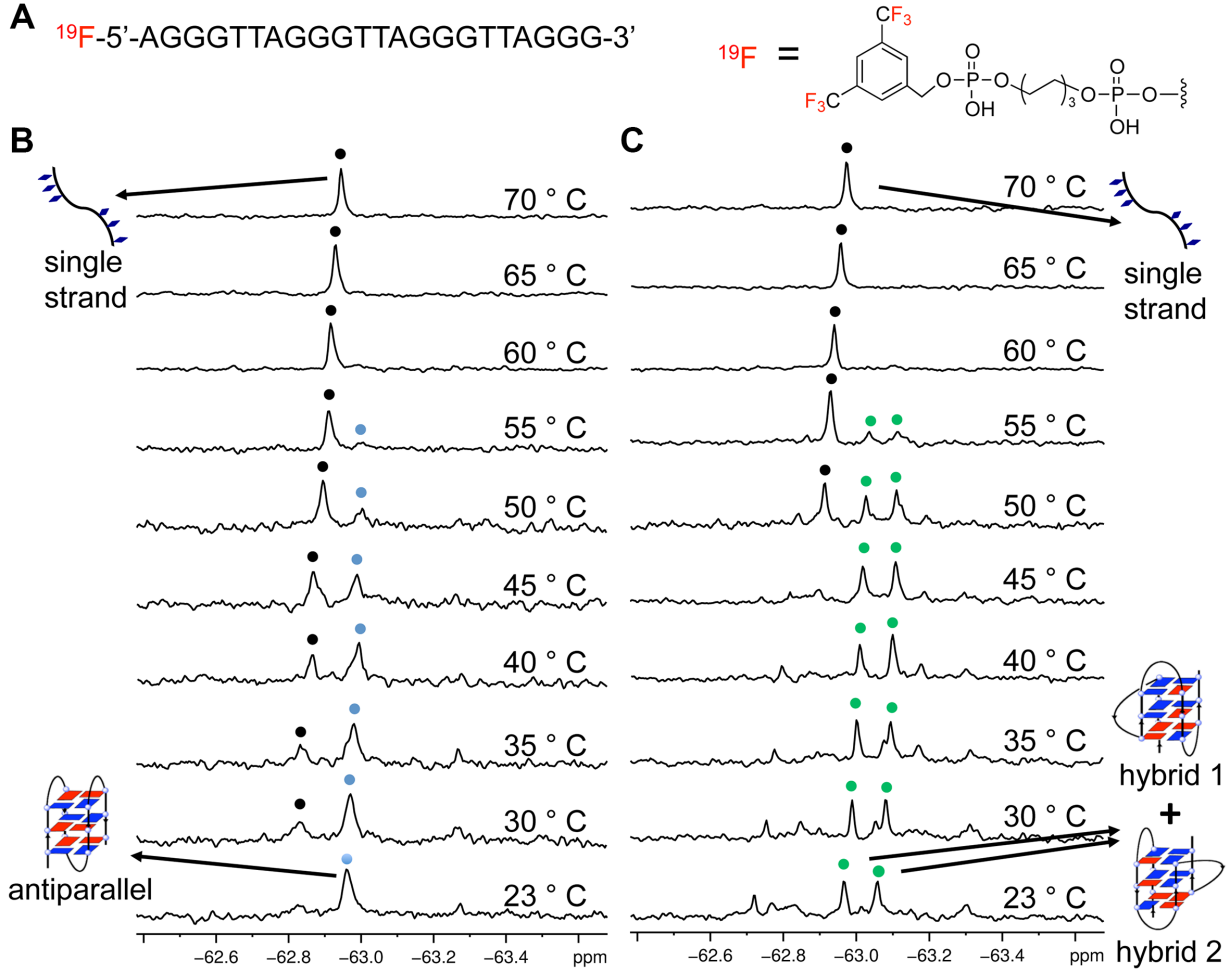
^19^F NMR spectra of ^19^F-labeled 22-mer ODN 1 d[AGGG(TTAGGG)_3_] in Na^+^ and K^+^ solutions. (**A**) Chemical structure of ^19^F-labeled DNA bearing ^19^F group at the 5′ terminal. (**B**)^19^F NMR of ^19^F-labeled DNA at different temperatures in Na^+^ solution. (**C**) ^19^F NMR of ^19^F-labeled DNA at different temperatures in K^+^ solution. Blue and black spots indicated antiparallel G-quadruplex and single strand, respectively. Green indicated hybrid-1 and hybrid-2 G-quadruplexes conformations. Temperatures indicated on the right. Condition: 0.1 mM DNA in (B) 300 mM NaCl and 20 mM Na-PO_4_ buffer (pH 7.0) or (C) 100 mM KCl and 20 mM K-PO_4_ buffer (pH 7.0). The sample is kept for 10 min of each temperature for ^19^F NMR detection.

To study different G-quadruplex structures, we introduced a 3,5-bis(trifluoromethyl)benzene moiety into the 5′ terminus of the 22-mer telomeric ODN 1 sequence (Figure 1A and Supplementary Figures S1–S6). The ^19^F modification can be applied in sugar ring, nucleic base and terminal of strands (33). All of the modifications are useful for studying nucleic acids structure, property and function. Recently, a ^19^F sensor at inside location of DNA strands was employed to investigate the telomere DNA structure in *Xenopus laevis* oocytes (24). Only one fluorine atom in the probe is hard to offer a high ^19^F NMR signal intensity and therefore requires a long time for in-cell measurement (8–10 h). On the other hand, advantageously, the ^19^F NMR sensor that has been inserted into the DNA could offer additional insights into the local structural change.

Terminal labeling is a very convenient method to connect the ^19^F NMR sensor and target oligonucleotide since it requires only one step of chemical synthesis. We choose the 3,5-bis(trifluoromethyl)benzene moiety as an external ^19^F labeling that has six equivalent ^19^F atoms and was expected to afford the high ^19^F NMR intensity. The labeling at 5′ end may prevent the enzymatic degradation of ^19^F label telomere DNA. These allowed us to more clearly observe the structure of telomere DNA in human cells.

We employed ^1^H NMR and CD spectroscopy to investigate the ^19^F-labeled DNA G-quadruplex structures. We observed similar imino proton and aromatic regions of ^1^H NMR in Na^+^ solutions for natural and ^19^F-labeled DNA (Supplementary Figure S7). In K^+^ solution, the modified sequence shows a slight difference in imino peaks from the native sequence. Comparison of 1D ^1^H NMR spectra may not be sufficient to prove or disprove similarity of G-quadruplex structure and other analysis methods would be needed, such as CD and 2D NMR spectra. The CD spectrum of ^19^F-labeled ODN 1 in the presence of Na^+^ at 20°C showed a negative band at 265 nm and a positive peak at 295 nm (Supplementary Figure S8), which is similar to the spectrum for the natural sequence, indicating the formation of antiparallel G-quadruplex. In K^+^ solution (an aqueous solution containing 100 mM KCl and 20 mM K-PO_4_ buffer), natural and ^19^F-labeled OND 1 showed a negative band at 240 nm and a positive band at 290 mm, indicating the formation of a hybrid-type G-quadruplex (Supplementary Figure S8). Furthermore, 2D NMR experiment is performed to confirm that ^19^F modification does not change the structure of G-quadruplex. We observed cross peaks from the imino proton region of 2D NOESY spectra and found that they are similar with the result of previous study (Supplementary Figure S9) (12).

Stronger background in the cellular environment often leads to poor-quality in-cell ^1^H NMR spectra. Because there is no natural intracellular concentration of fluorine in cells, there is no background noise in in-cell ^19^F NMR spectra. Therefore, ^19^F NMR spectroscopy is an ideal tool for studying G-quadruplex structures in living cells. However, ^19^F label of oligonucleotide is required when using ^19^F NMR. Such as a label may affect the folding of the DNA G-quadruplex. Although the label may influence G-quadruplex formation, the approach, and so far, tend to work effectively and provide valuable information for understanding the structures of nucleic acid in cells. In the future, the more effective approaches for probing nucleic acid structures in cells are desired and need to be further developed.

A ^19^F NMR experiment was performed to investigate the ODN 1 in Na^+^ solution (an aqueous solution containing 300 mM NaCl and 20 mM Na-PO_4_ buffer). One sharp peak at −62.57 ppm was observed, which is consistent with the ^1^H NMR and CD results and with a previous report that showed the ^19^F-labeled ODN 1 sequence could form an antiparallel G-quadruplex in Na^+^ solution (Figure 1B) (12). As the temperature increased, the intensity of the signal decreased; upon heating to 50°C, a new peak corresponding to the unfolded single strand appeared, and at 75°C, only this peak remained with a strong intensity.

In the K^+^ solution, two major peaks at −62.97 and −63.06 ppm were observed (Figure 1C), which are characterized as hybrid-1 and hybrid-2 G-quadruplex conformations according to the previous studies (13–16). Similarly, upon heating the sample, a clearly two-state structural transition from hybrid-type G-quadruplex conformations to a single strand was observed. In addition, we note that several small signals around the two major peaks were observed while they disappeared at higher temperature (60°C), which may suggest the existence of other intermediates and G-quadruplex conformations, such as G-triplex, G-hairpin and two-tetrad G-quadruplex (19–21).

**Figure 2.**
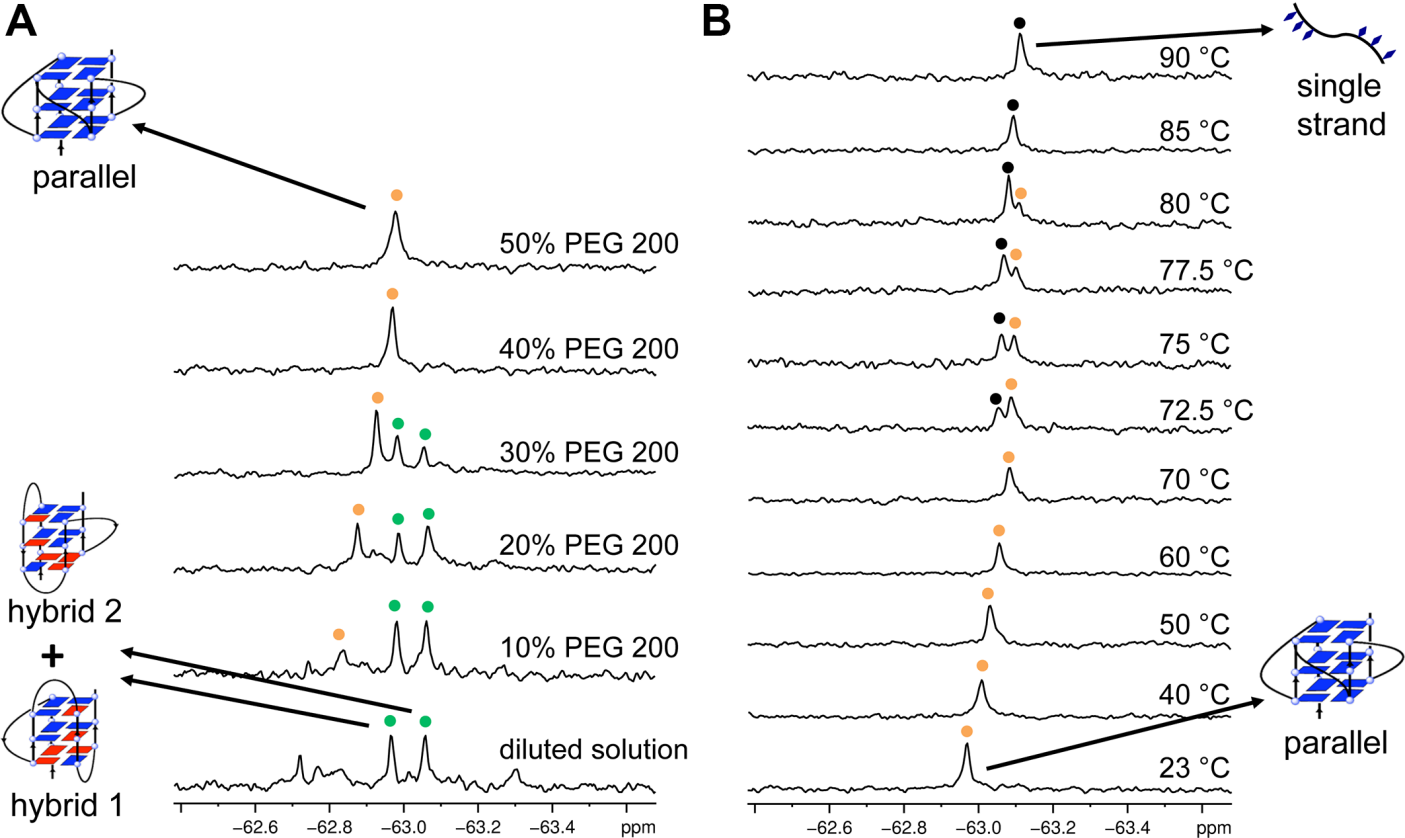
^19^F NMR spectra of ^19^F-labeled ODN 1 in PEG 200. (**A**) ^19^F NMR of ^19^F-labeled 22-mer DNA at different concentration of PEG 200 in K^+^ solution. (**B**) ^19^F NMR of ^19^F-labeled ODN 1 at different temperatures in 40% PEG 200. Green indicated hybrid-1 and hybrid-2 G-quadruplexes conformations. Orange and black spots indicated parallel G-quadruplex and single strand, respectively. PEG 200 ratio and temperatures indicated on the right. Condition: 0.1 mM DNA in 100 mM KCl and 20 mM K-PO_4_ buffer (pH 7.0). The sample is kept for 10 min of each temperature for ^19^F NMR detection.

It has been reported that PEG 200 can induce the structural transition from a hybrid-type to a parallel G-quadruplex (18). Using ^19^F NMR spectroscopy, we monitored the transition process from the hybrid-type to the parallel G-quadruplex (Figure 2A). With the addition of 10% (v/v) PEG 200 to the diluted solution, a new peak at −62.83 ppm appeared, and its intensity became remarkably greater than that of the hybrid-type G-quadruplex at 30% PEG 200. The original peaks for hybrid-type G-quadruplex completely disappeared at 40% and 50% PEG 200. The ^19^F NMR results are consistent with previous research that PEG 200 can induce the structural transition from a hybrid-type to a parallel G-quadruplex (18). Therefore, we assigned the new peak as parallel G-quadruplex conformation. Figure 2B shows that as the temperature increased from 23 to 90°C in crowded solution of 40% (v/v) PEG, a new peak corresponding to the unfolded single strand appeared and, at 85°C, only this peak remained with a strong intensity. CD experiments were also performed to confirm the formation of parallel G-quadruplexes under molecular crowding mimics (Supplementary Figure S10).

We further labeled the sequence ODN 2 d[GGG(TTAGGG)_3_T] of two-tetrad antiparallel G-quadruplex with ^19^F sensor and compared its conformation with natural sequence. CD and ^1^H NMR showed that ODN 2 formed a two-tetrad G-quadruplex conformation consistent with previous studies (Supplementary Figures S11 and S12) (19–21). Although imino NMR signals of ^19^F labeled and natural ODN2 are not completely similar to each other (Supplementary Figure S12), the eight imino peaks observed from ^19^F-labeled ODN2 indicate the formation of a two-tetrad G-quadruplex. Several small peaks in imino region were observed for both ^19^F labeled and natural ODN 2 sequences, which is consistent with the previous study that ODN 2 also could form same minor G-quadruplex conformations (20). We further carried out the ^19^F NMR experiment to investigate the structure of the ^19^F-labeled ODN 2 in various conditions (Supplementary Figure S13). In K^+^ solution, one major peak at −62.59 ppm was observed with several small signals in the downfield, which is consistent with the CD and imino proton NMR results and previous study, that OND 2 could majorly form a two-tetrad antiparallel G-quadruplex conformation (20). With increasing the temperature to 70°C, the peak at −62.59 ppm disappeared and a new signal for single strand at −62.79 ppm appeared. Furthermore, the addition of 40% PEG 200 to the K^+^ solution at room temperature resulted in a peak shift from −62.59 to −63.08 ppm, which indicated the formation of parallel G-quadruplex induced by PEG 200.

*T*_m_ values and thermodynamic parameters for each G-quadruplex were determined by ^19^F NMR spectra (Table 1 and Supplementary Figure S14). In K^+^ solution, using ^19^F NMR spectroscopy, we could simultaneously monitor the unfolding progress of two different types of hybrid G-quadruplexes and obtain the *T*_m_ values and thermodynamic parameters, which is not easy to do using other methods. The high *T*_m_ values of various G-quadruplexes suggested stable G-quadruplex formation. The two-tetrad G-quadruplex is more stable than the three layers hybrid G-quadruplex in K^+^ solution consistent with previously reported (19–21). We further investigated the effect of the ^19^F-labeling on the stability of the G-quadruplex structures by CD melting experiment. ^19^F NMR could not be applied to study the thermodynamic stability of natural DNA sequence. Thus, we performed CD melting experiment to obtain the *T*_m_ values for each natural and ^19^F-labeled G-quadruplex topology. As shown in Supplementary Table S1, the *T*_m_ values for each G-quadruplex are most same or slightly decreased with the fluorine modification.

**Table 1.** The thermodynamic parameters were determined from van’t Hoff plots

G-quadruplex conformations	−Δ*H* (kJ/mol)	−Δ*S* (J/mol K)	−Δ*G_298_* (kJ/mol)	*T*_m_ (°C)
Antiparallel	130.5	410.2	7.4	45.4
Two-tetrad	189.6	569.4	19.9	59.9
Hybrid-1 /hybrid-2	174.6/166.8	537.2/514.2	14.5/13.6	52.7/51.8
Parallel	263.4	755.6	38.2	75.6

Detail procedure was reported in our previous reference (26). The experimental errors for enthalpy (Δ*H*) and entropy (Δ*S*) were ± 5 kJ/mol and ± 10 J/mol K, respectively.

### ^19^F NMR analysis of ^19^F labeled telomeric DNA G-quadruplexes in living HeLa cells

**Figure 3.**
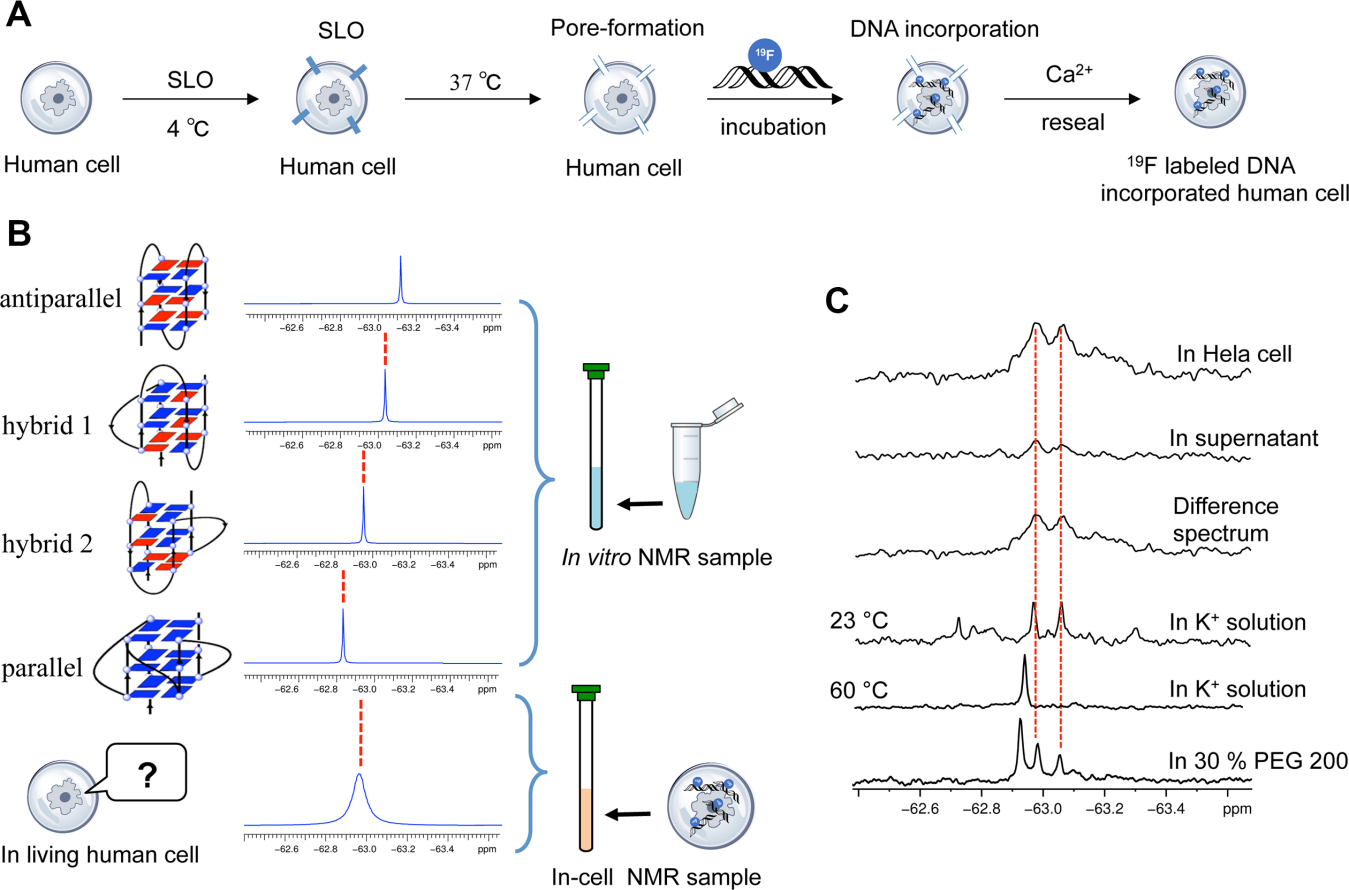
In-cell ^19^F NMR of ^19^F labeled ODN 1. (**A**) Schematic overview of the SLO treatment cell system for transfection DNA into HeLa cells. (**B**) Comparison with the position of reference *in vitro* spectrum provides a reliable determination of intracellular ^19^F-labeled DNA. (**C**) Comparison of ^19^F NMR spectra of ODN 1 in K^+^ solution, in HeLa cell, in supernatant, difference spectrum between HeLa cell and supernatant, in K^+^ solution (23 and 60°C) and in 30% PEG 200.

Encouraged by the ability to use ^19^F NMR spectroscopy to monitor the conformation of DNA G-quadruplex, we utilized ^19^F NMR to detect telomeric DNA G-quadruplex in HeLa cells. Unlike direct injection of relatively large size *Xenopus laevis* oocytes, microinjection of HeLa cells is not possible due to the small size of human cells (34–37). Therefore, we utilized a new method using streptolysin O (SLO) to transfect ^19^F-labeled DNA into HeLa cells (Figure 3A) (32,38). SLO can bind cholesterol in the plasma membrane at low temperature (4°C) and form pores in the membrane to allow the permeabilization of biomolecules into cells at high temperature (37°C). The pore formed by SLO can be resealed by the addition of Ca^2+^. The SLO treatment cell system can be used as a type of cellular test tube to study the structures, properties and functions of biomolecules. Here, we transfected ^19^F-labeled 22-mer telomeric ODN 1 into HeLa cells by using the SLO treatment system for in-cell ^19^F NMR measurements. We examined the transfection efficiency of the FAM-labeled DNA into HeLa cells using confocal fluorescence microscopy and flow cytometry analysis. Green fluorescence in HeLa cells was clearly observed with the treatment of SLO (Supplementary Figure S15), which suggested the successful transfection of DNA into HeLa cells. In the flow cytometry experiment, the cells exhibited increased FAM positive and PI-negative populations (55%), indicating that pores formed and resealed properly in cells (Supplementary Figure S16) consistent with previous reports (32,38). To compare the in-cell ^19^F NMR spectrum with the *in vitro* results of ^19^F NMR signals for different G-quadruplex conformations, antiparallel, parallel, hybrid-1 and hybrid-2, a reliable determination of the telomeric DNA G-quadruplex conformation in living human cells is required (Figure 3B). In-cell ^19^F NMR results showed two major peaks at approximately −62.97 and −63.06 ppm, which were almost identical to those observed in the K^+^ solution. Thus, the in-cell ^19^F NMR spectrum demonstrates that ^19^F-labeled telomeric DNA can present hybrid-1 and hybrid-2 G-quadruplex structures in living human cells. Our result is different from a recent report indicating that only the hybrid-2 G-quadruplex is observed in *Xenopus laevis* oocytes (24). This structural difference between *Xenopus laevis* oocytes and human cells might be related to the preference of cells from a marked species and the difference in the ability to form higher order DNA structures. A report suggested that parallel G-quadruplex structure is not preferred in *Xenopus laevis* oocyte extraction (39). Here, we confirmed the formation of two different hybrid G-quadruplexes in living human cells for the first time.

After the NMR measurement, the outer solution of the cell suspension was collected and examined by ^19^F NMR spectroscopy. Almost no signal was observed from the supernatant, indicating that almost all of the NMR signals originated from the ^19^F-labeled DNA within the HeLa cells. We produced a difference spectrum between the HeLa cells and the suspension to eliminate the signal from the supernatant. The clear signal in the difference spectrum supports the observation of two hybrid-type G-quadruplex structures in living human cells.

To confirm that the ^19^F NMR chemical shift of different DNA G-quadruplex topologies are identical in both *in vitro* and *in vivo* conditions, we performed ^19^F NMR experiment of ^19^F-labeled telomeric ODN 1 in HeLa cell extract, which offers an *ex vivo*-like context. As result shown in Supplementary Figure S17, the ^19^F NMR signals in HeLa cell extract are almost same with that in K^+^ solution and in living HeLa cells. We note that several small peaks disappeared in HeLa lysate. We speculate that the *ex vivo* molecular crowding environment may influence the hybrid G-quadruplex structure. Furthermore, an imino proton region NMR spectrum was measured in HeLa cell extract (Supplementary Figure S18). The *ex vivo* NMR spectrum has a lower resolution compared to sample in dilute solution due to the high viscosity of the cell extract and inherent sample inhomogeneity (35). The peaks positions and intensities in *ex vivo* NMR may be similar to the NMR signal obtained from K^+^ solution. Thus, the ^1^H NMR results can be used as useful evidence to support the interpretation in terms of what happens in living cells by ^19^F NMR, which also reported in other papers (21,24,39). We also monitor ^19^F-labeled telomeric DNA G-quadruplex structure in HeLa cell extract with different lengths of time. As results shown in Supplementary Figure S17, even incubated with HeLa cell extract for 6 h, the ^19^F NMR signals did not change, suggested that the ^19^F-labeled DNA G-quadruplex is stable in a cellular environment during in-cell NMR measurement time scale (1 h).

In ODN 2, only one ^19^F NMR signal was observed after incorporation into HeLa cells, which chemical shift is same with the peak obtained from two-tetrad G-quadruplex in K^+^ solution (Supplementary Figure S13). Our results indicated that telomere DNA sequence could adopt the two-tetrad G-quadruplex conformation in living human cells.

To assess the generality and reliability of the ^19^F NMR approach to study telomeric DNA structures, we performed ^19^F NMR experiment of a 25-mer telomeric DNA d[TAGGG(TTAGGG)_3_TT] ODN 3 *in vitro* and in living human cells (Supplementary Figures S19–S22). As shown in Supplementary Figure S19, one major peak was observed, which is consisted with the previous report that the 25-mer telomeric DNA sequence could majorly formed a hybrid-2 G-quadruplex in K^+^ solution (40,41). With the addition of 40% PEG 200 to the DNA solution, a new peak appeared, which indicated the structural transition from hybrid G-quadruplex to parallel G-quadruplex (Supplementary Figure S20), which has been also confirmed by the CD results (Supplementary Figure S21). One major peak and one minor peak were observed in the HeLa cell extract with the same chemical shift as observed in the K^+^ solution, which suggested that the 25-mer telomeric DNA sequence can majorly form a hybrid-2 G-quadruplex in an *ex vivo*-like context (Supplementary Figure S22). In HeLa cells, we observed a broad peak with an apparent maxima centered at −62.83 ppm (Supplementary Figure S22), which chemical shift is identical with the hybrid-2 G-quadruplex signal observed in K^+^ solution, indicating that the 25-mer telomeric DNA can form a hybrid-2 G-quadruplex structure in living human cells.

The two 4-repeat sequences used in this study were well defined in previous reports (13–16, 40,41), which ODN 1 can form two hybrid G-quadruplex while ODN 3 only form hybrid-2 G-quadruplex structure in K^+^ solution. Thus, we could use ^19^F NMR to compare their different structural behaviors *in vitro* and living human cells. Compared to 22-mer telomeric DNA ODN 1, 25-mer telomeric DNA ODN 3 has the additional dT nucleosides in two terminals. The additional nucleosides shift the 25-mer telomeric DNA sequence to form a stable hybrid-2 G-quadruplex in K^+^ solution and in HeLa cells, which is different to 22-mer telomeric DNA forming both of two hybrid-type G-quadruplexes (hybrid-1 and hybrid-2). Therefore, these results indicated that the different nucleosides at the terminals of telomere DNA sequences are essential for their formation of different G-quadruplex topologies.

In conclusion, using ^19^F NMR, we could directly distinguish the parallel, antiparallel, hybrid-1 and hybrid-2 G-quadruplex structures and determine the thermodynamic properties of the different types of G-quadruplexes. Importantly, in-cell ^19^F NMR demonstrated, for the first time, that the telomeric DNA sequence forms two hybrid-type and two-tetrad antiparallel G-quadruplex structures in living human cells. This result provides valuable information for understanding the structures of human telomeric DNA in living human cells.

## Supplementary Material

gkz276_Supplemental_File
