# Supplementary material for: Hybrid-type and two-tetrad antiparallel telomere DNA G-quadruplex structures in living human cells
Source: Nucleic Acids Res. 2019 Apr 12;47(10):4940–7. doi: 10.1093/nar/gkz276 (PMC6547409; doi:10.1093/nar/gkz276)
Supplement: gkz276_Supplemental_File [file gkz276_supplemental_file.pdf]

Supporting information

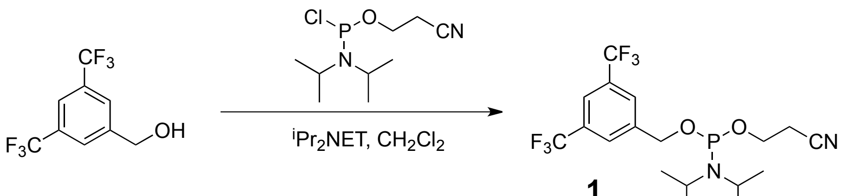

Supplementary Figure S1. Synthetic scheme of ^{19}F sensors compound 1 using phosphoramidite chemistry.

1-*O*-[(2-cyanoethoxy)-(*N*,*N*-diisopropylamino)phosphinyl]-3,5-bis(trifluoromethyl)benzene (1)

The 3,5-bis(trifluoromethyl)benzyl alcohol (960 mg, 3.9 mmol) was was dried three times with 5 mL anhydrous acetonitrile. The dried residues was treated with dry *N*,*N*-diisopropyl ethylamine (1 mL, 7.4 mmol) and 2-cyanoethyl-*N*,*N*- diisopropylchlorophosphoramidite (1 mL, 4.3 mmol) in dry dichloromethane (5 mL) and stirred at room temperature for 2 h. The reaction was stopped by adding 5% $NaHCO_3$ aqueous solution (50 ml). After addition of dichloromethane (50 ml), the aqueous layer was extracted three times with dichloromethane (50 ml). The combined organic layers were dried over by Na_2SO_4 and the solvent was evaporated *in vacuum*. The residue was purified via recycling preparative HPLC to give the compound **1** (1.2 g, 2.7 mmol, 69%). ^{1}H-NMR (400 MHz, $CDCl_3$) δ 7.81 (s, 2H), 7.79 (s, 1H), 4.84 (m, 2H), 3.95-3.79 (m, 2H), 3.67 (m, 2H), 2.66 (t, J = 6.3 Hz, 2H), 1.21 (m, 12H). ^{13}C-NMR (100 MHz, $CDCl_3$) δ 141.94, 141.87, 131.63 (q, J = 33 Hz), 126.79, 123.33 (q, J = 271 Hz), 121.29 117.45, 64.34, 64.15, 58.52, 58.33, 43.39, 43.27, 24.59 (t, J = 8 Hz), 20.44, 20.37. ^{31}P-NMR (162 MHz, $CDCl_3$) δ −149.61. ESI-MS for $C_{18}H_{24}O_2N_2F_6P$ $[M+H]^+$: Calcd. 445.1474; Found. 445.1471.

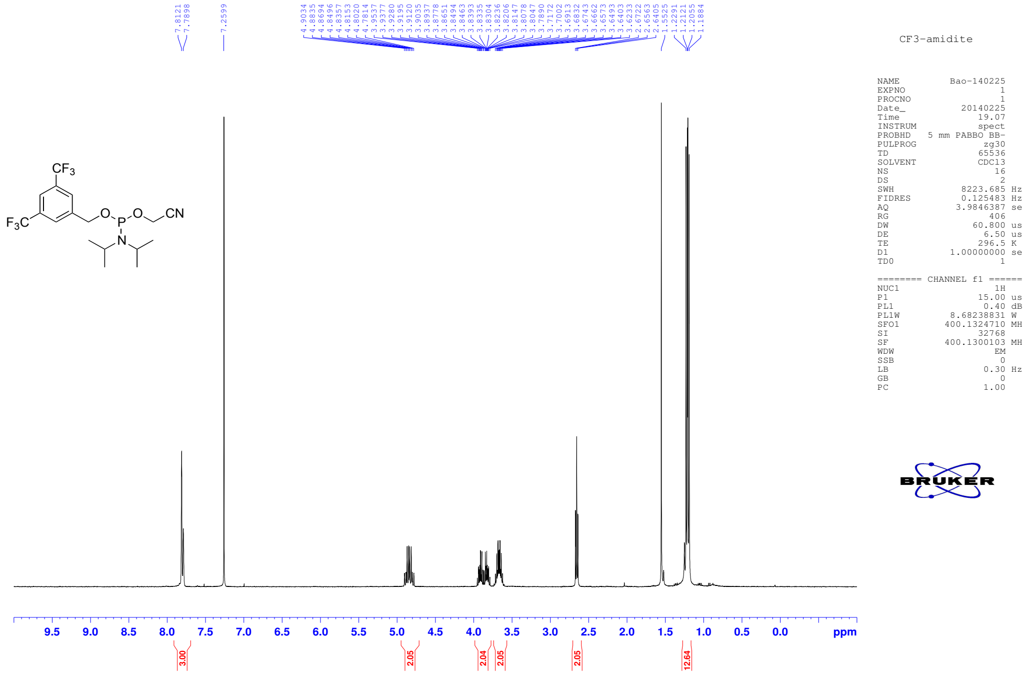

Supplementary Figure S2. ^{1}H NMR spectrum of compound 1.

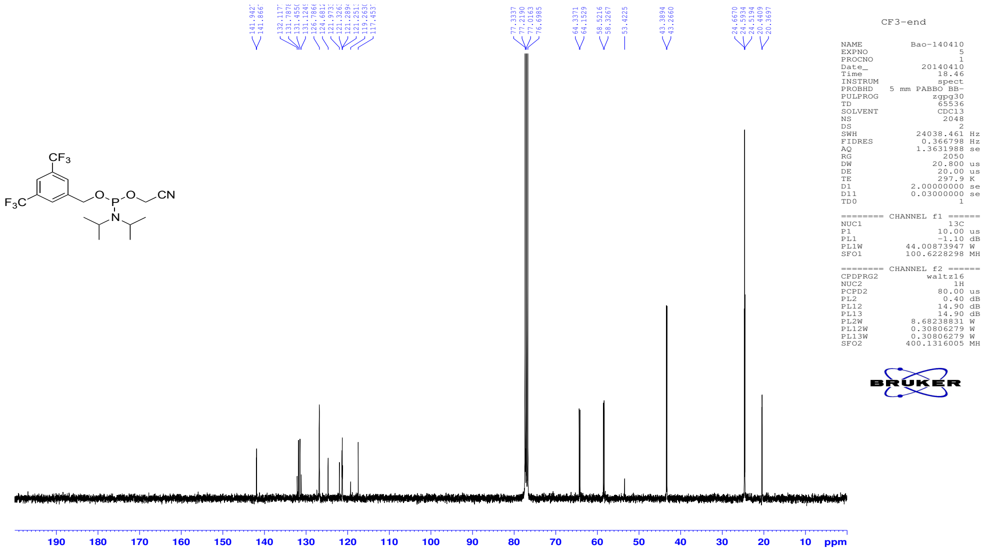

Supplementary Figure S3. ^{13}C NMR spectrum of compound 1.

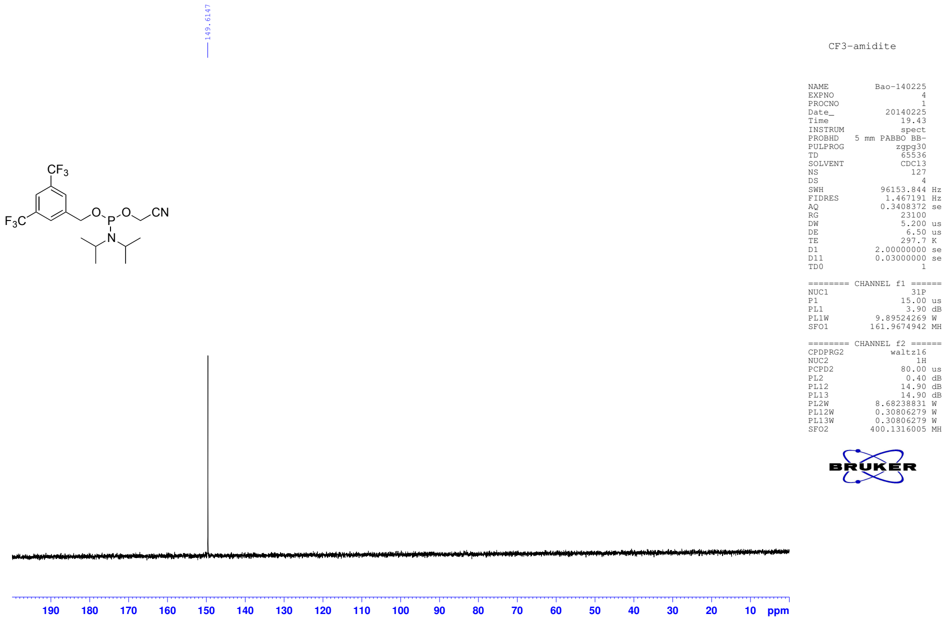

Supplementary Figure S4. ^{31}P NMR spectrum of compound 1.

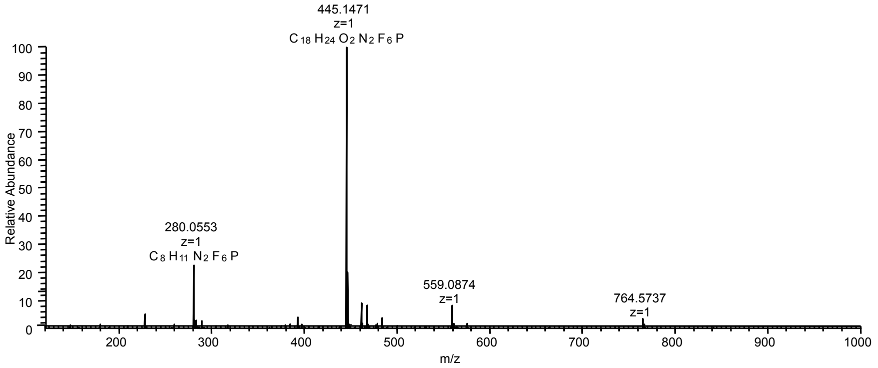

Supplementary Figure S5. ESI-MS spectrum of compound 1.

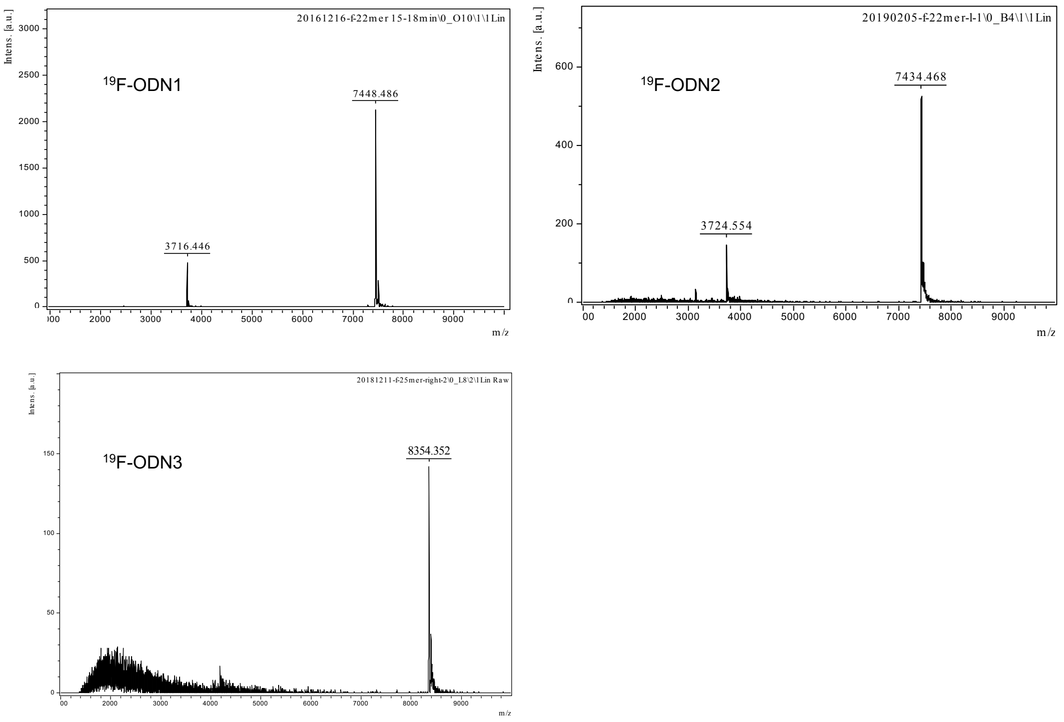

Supplementary Figure S6. MALDI-TOF MS of ^{19}F labeled telomeric DNA sequences.

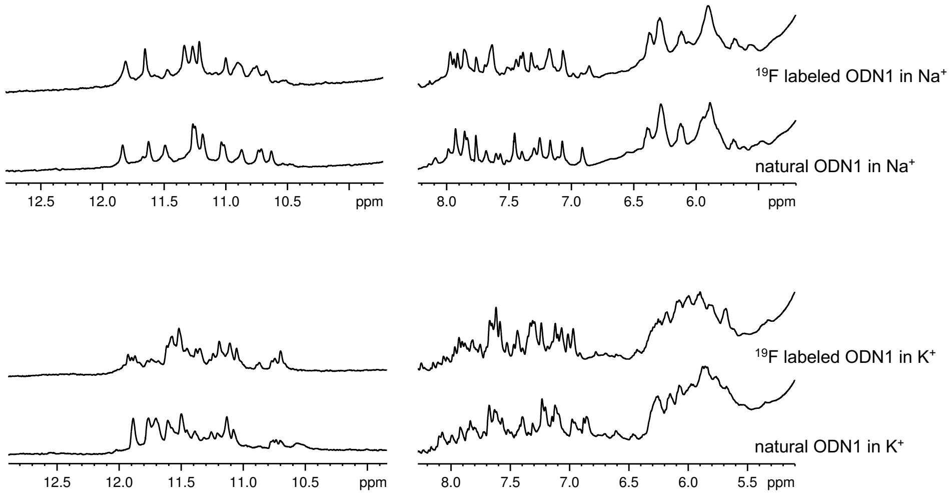

Supplementary Figure S7. ^{1}H NMR spectra of ^{19}F labeled and natural ODN 1. 0.5 mM DNA in 300 mM NaCl and 20 mM Na-PO_4 or 100 mM KCl and K-PO_4 buffer (pH 7.0). The ^{1}H NMR spectra of ^{19}F labeled DNA is very similar to that of natural DNA, suggesting ^{19}F sensor does not induce the conformation change of DNA G-quadruplex.

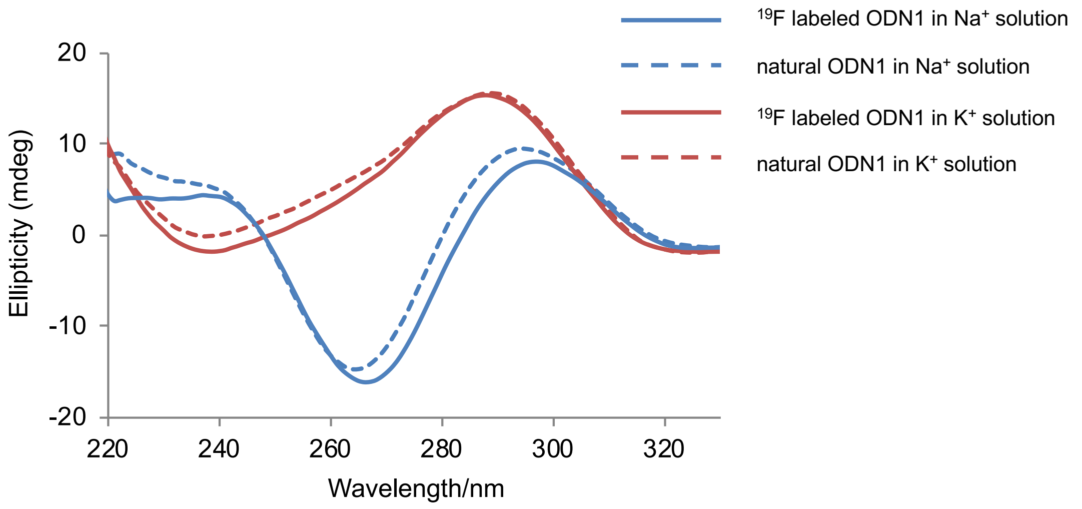

Supplementary Figure S8. CD spectra of ^{19}F labeled and natural ODN 1. 5 μM DNA in 100 mM KCl (red) or NaCl (blue) and 20 mM K-PO_4 or Na-PO_4 buffer (pH 7.0). The CD spectra of ^{19}F labeled DNA is very similar to that of natural DNA, suggesting ^{19}F sensor does not induce the conformation change of DNA G-quadruplex.

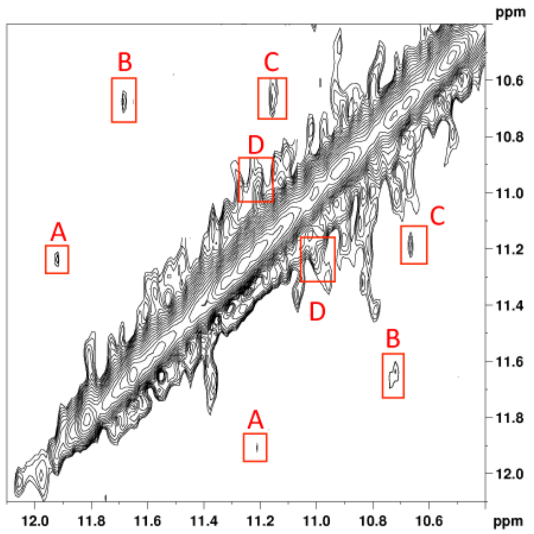

Supplementary Figure S9. H1-H1 imino proton region 2D-NOESY spectrum of ^{19}F labeled ODN 1 in the presence of 200 mM NaCl and 10 mM Na-phosphate. A, B, C, and D peaks were similar with that observed in the reference paper (1) and suggested ^{19}F modification does not change the structure of antiparallel G-quadruplex.

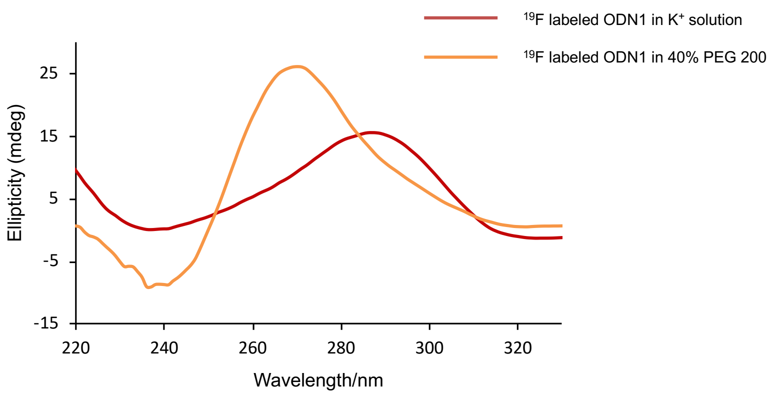

Supplementary Figure S10. CD spectra of ^{19}F labeled ODN 1. Condition: 5 μM DNA, 100 mM KCl and 20 mM K-PO_4 (pH 7.0) in the presence or absence of 40% PEG 200.

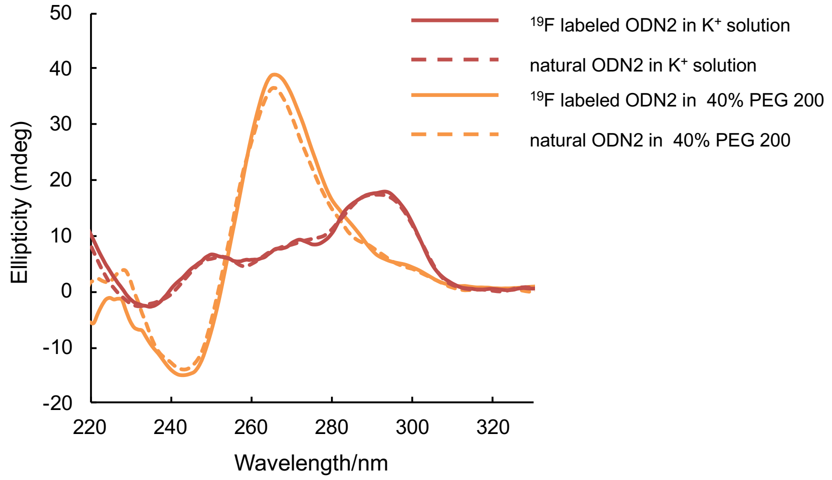

Supplementary Figure S11. CD spectra of ^{19}F labeled and natural ODN 2. 5 μM DNA, 100 mM KCl 20 mM K-PO_4 (pH 7.0) in the absence (red) or presence (yellow) of 40% PEG 200. The CD spectra of ^{19}F labeled DNA is very similar to that of natural DNA, suggesting ^{19}F sensor does not induce the conformation change of DNA G-quadruplex.

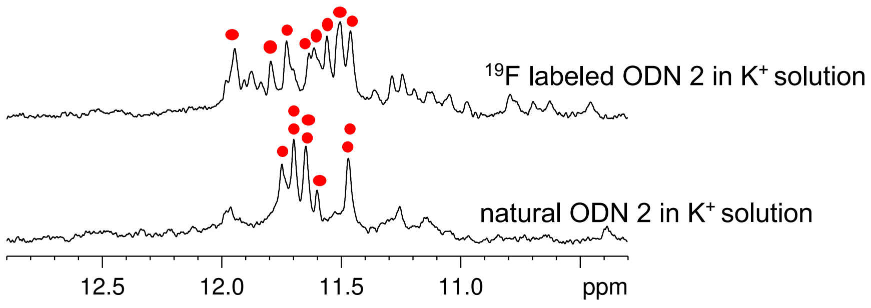

Supplementary Figure S12. ^{1}H NMR spectra of ^{19}F labeled and natural ODN 2. 0.5 mM DNA in 100 mM KCl and 20 mM K-PO_4 (pH 7.0). Eight imino peaks observed from ^{19}F labeled ODN2 indicate the formation of a two-tetrad G-quadruplex.

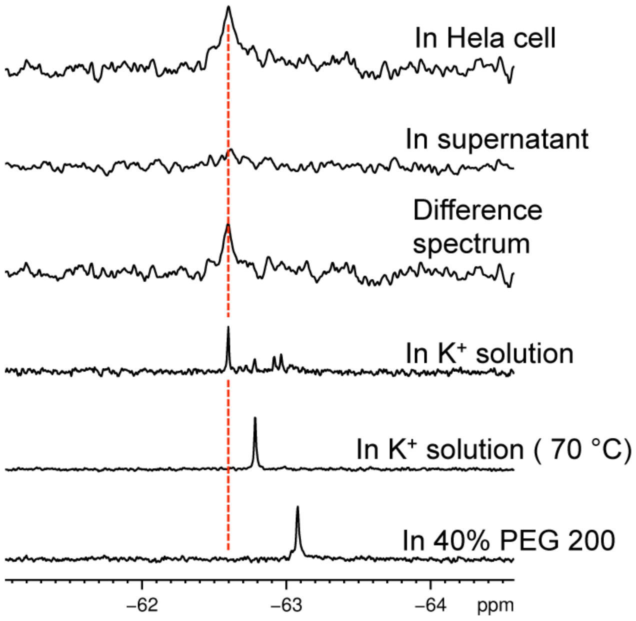

Supplementary Figure S13. ^{19}F NMR of ^{19}F labeled ODN 2 in various conditions. For *in vitro* experiments condition: 0.1 mM DNA in 100 mM KCl and 20 mM K-PO4 buffer (pH 7.0).

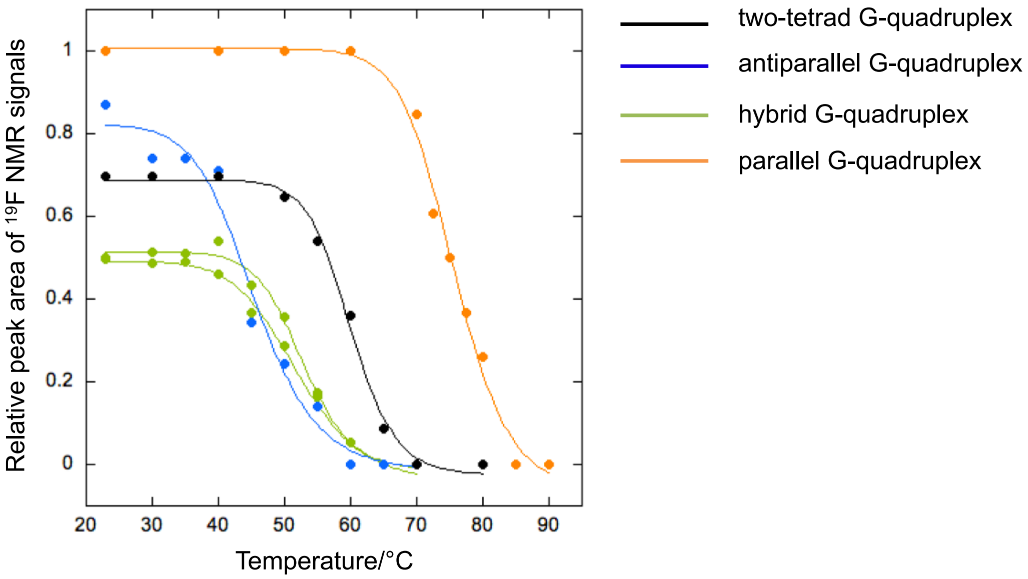

Supplementary Figure S14. Profiles of the relative peak areas of the ^{19}F resonance signals versus temperature.

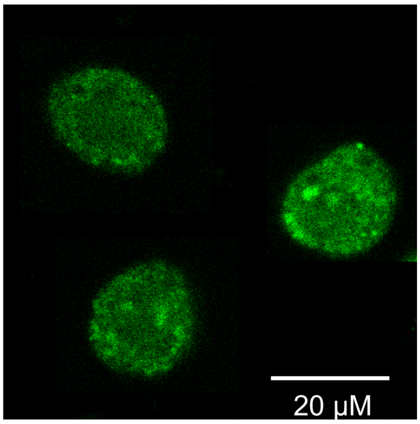

Supplementary Figure S15. Confocal microscopy images of SLO-treated HeLa cells with FAM-labeled DNA.

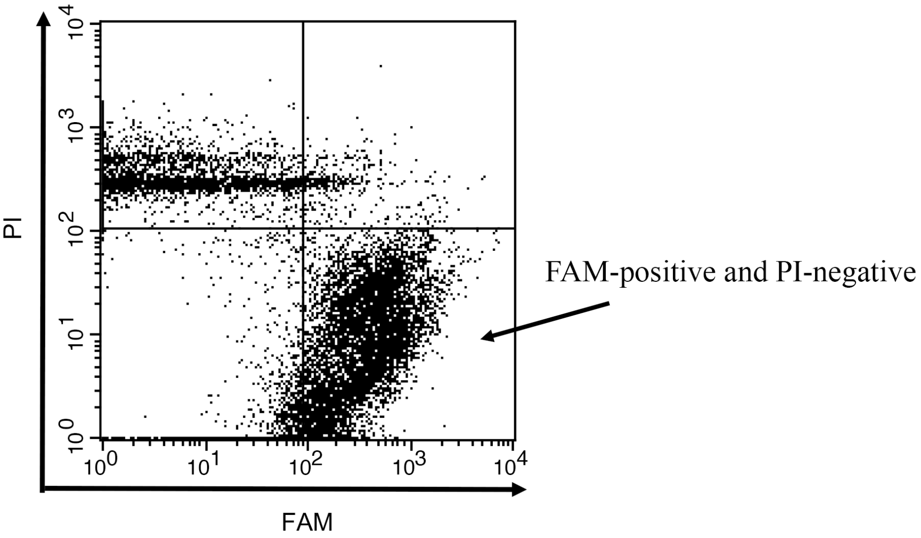

Supplementary Figure S16. FCM analysis of SLO-treated HeLa cells with FAM-labeled DNA and PI. FAM-positive and PI-negative populations as living cells were indicted.

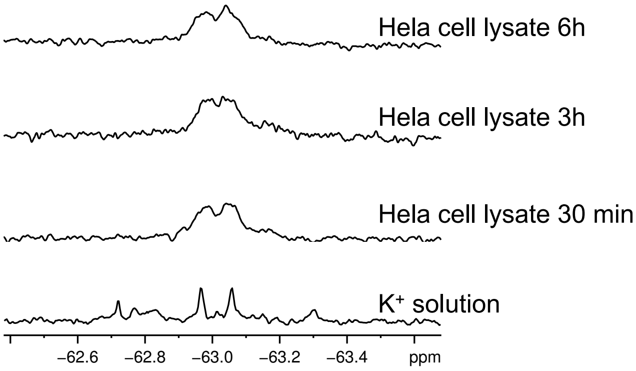

Supplementary Figure S17. ^{19}F NMR spectra of ^{19}F labeled ODN 1 in Hela cell lysate with different length of time. Even incubated with Hela cell lysate for 6 h, the ^{19}F NMR signals did not change, suggested that the ^{19}F labeled DNA G-quadruplex is stable in a cellular environment during in-cell NMR measurement time scale (1h).

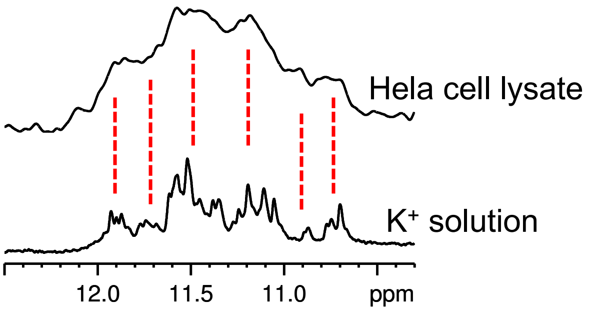

Supplementary Figure S18. Imino NMR spectra of ^{19}F labeled ODN 1 in K^+ solution and in Hela cell lysate. The ex-vivo NMR spectrum has lower resolution compared to sample in dilute solution due to the high viscosity of the cell extract and inherent sample inhomogeneity (2). The peaks positions and intensities in ex-vivo NMR may be similar to the NMR signal obtained from K^+ solution. Thus, the ^{1}H NMR results can be used as useful evidence to support the interpretation in terms of what happens in living cells by ^{19}F NMR, which also reported in other papers (3-5).

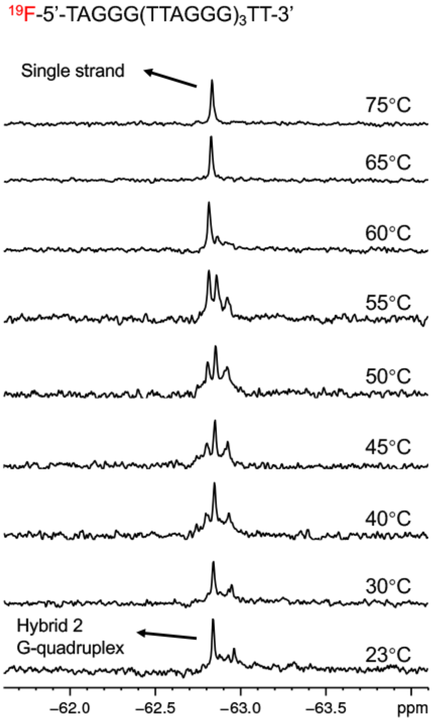

Supplementary Figure S19. ^{19}F NMR of ^{19}F labeled ODN 3 at different temperatures in K^+ solution. Condition: 0.1 mM DNA in 100 mM KCl and 20 mM $K\text{-}PO_4$ buffer (pH 7.0). The sample is kept for 10 min of each temperature for ^{19}F NMR detection.

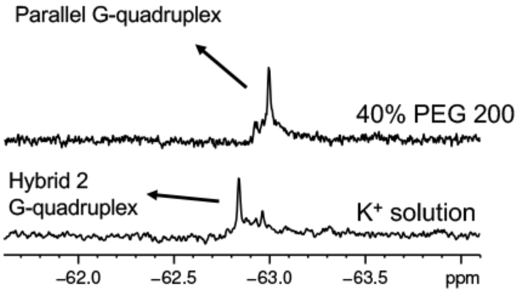

Supplementary Figure S20. ^{19}F NMR of ^{19}F labeled ODN 3 in the presence or absence of 40% PEG 200. Condition: 0.1 mM DNA in 100 mM KCl and 20 mM $K\text{-}PO_4$ buffer (pH 7.0).

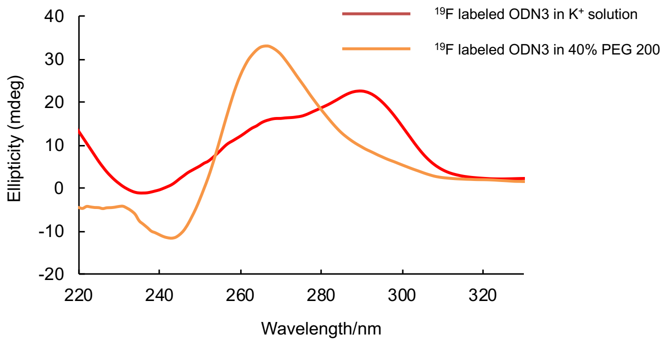

Supplementary Figure S21. CD spectra of ^{19}F labeled ODN 3. Condition: 5 μM DNA, 100 mM KCl and 20 mM K-PO4 (pH 7.0) in the presence or absence of 40% PEG 200.

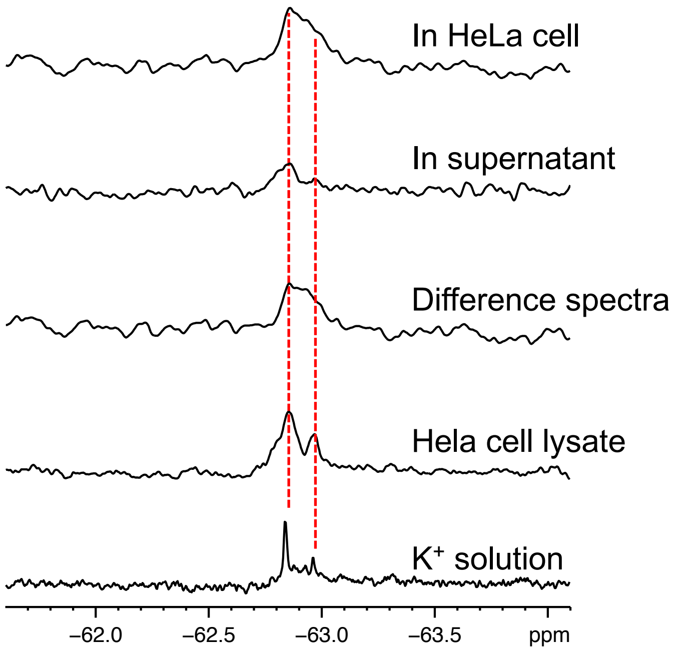

Supplementary Figure S22. Comparison of ^{19}F NMR spectra of ODN 3 in K^+ solution, in Hela cell lysate, in HeLa cell, in supernatant and difference spectrum between HeLa cell and supernatant.

Table S1. T_m values of ^{19}F labeled and natural telomeric DNA determined by CD melting experiment

sequence	G-quadruplex conformation	T_m (°C)
[19]F ODN1	antiparallel	48.2
	hybrid	58.2
	parallel	75.1
Natural ODN1	antiparallel	49.7
	hybrid	58.5
	parallel	76.1
[19]F ODN2	two-tetrad antiparallel	62.8
	parallel	81.8
Natural ODN2	two-tetrad antiparallel	62.8
	parallel	81.9

Reference:

1. Wang, Y.; Patel, D. J. (1993) Solution Structure of the Human Telomeric Repeat d[$AG_3(T_2AG_3)_3$] G-Tetraplex. *Structure.* 1, 263-282.
2. Hansel, R.; Foldynova-Trantirkova, S.; Lohr, F.; Buck, J.; Bongartz, E.; Bamberg, E.; Schwalbe, H.; Dotsch, V.; Trantirek, L. (2009) Evaluation of Parameters Critical for Observing Nucleic Acids inside Living Xenopus laevis Oocytes by In-Cell NMR Spectroscopy. *J. Am. Chem. Soc.*, **131**, 15761-15768.
3. Hansel, R., Lohr, F., Trantirek, L. and Dotsch, V. (2013) High-resolution insight into G-overhang architecture. *J. Am. Chem. Soc.,* **135**, 2816-2824.
4. Manna, S.; Sarkar, D.; Srivatsan, S. G. (2018) A Dual-App Nucleoside Probe Provides Structural Insights into the Human Telomeric Overhang in Live Cells. *J. Am. Chem. Soc.,* **140,** 12622-12633.
5. Hansel, R.; Lohr, F.; Foldynova-Trantirkova, S.; Bamberg, E.; Trantirek, L.; Dotsch, V. (2011) The Parallel G-quadruplex Structure of Vertebrate Telomeric Repeat Sequences is Not the Preferred Folding Topology under Physiological Conditions. *Nucleic Acids Res.*, **39**, 5768-5775.
